# Begin at the beginning: A BAC-end view of the passion fruit (*Passiflora*) genome

**DOI:** 10.1186/1471-2164-15-816

**Published:** 2014-09-26

**Authors:** Anselmo Azevedo Santos, Helen Alves Penha, Arnaud Bellec, Carla de Freitas Munhoz, Andrea Pedrosa-Harand, Hélène Bergès, Maria Lucia Carneiro Vieira

**Affiliations:** Departamento de Genética, Universidade de São Paulo, Escola Superior de Agricultura “Luiz de Queiroz”, P.O. Box 83, 13400-970 Piracicaba, Brazil; INRA/Centre National de Ressources Génomiques Végétales, 24 Chemin de Borde Rouge, Auzeville CS 52627, 31326 Castanet Tolosan Cedex, France; Departamento de Botânica, Universidade Federal de Pernambuco, 50670-420 Recife, Brazil

**Keywords:** *Passiflora*, Passion fruit, Genomics, BAC-end sequencing, Repetitive elements, Gene content, Microsynteny, Fluorescent *in situ* hybridization

## Abstract

**Background:**

The passion fruit (*Passiflora edulis*) is a tropical crop of economic importance both for juice production and consumption as fresh fruit. The juice is also used in concentrate blends that are consumed worldwide. However, very little is known about the genome of the species. Therefore, improving our understanding of passion fruit genomics is essential and to some degree a pre-requisite if its genetic resources are to be used more efficiently. In this study, we have constructed a large-insert BAC library and provided the first view on the structure and content of the passion fruit genome, using BAC-end sequence (BES) data as a major resource.

**Results:**

The library consisted of 82,944 clones and its levels of organellar DNA were very low. The library represents six haploid genome equivalents, and the average insert size was 108 kb. To check its utility for gene isolation, successful macroarray screening experiments were carried out with probes complementary to eight *Passiflora* gene sequences available in public databases. BACs harbouring those genes were used in fluorescent *in situ* hybridizations and unique signals were detected for four BACs in three chromosomes (*n* = *9*). Then, we explored 10,000 BES and we identified reads likely to contain repetitive mobile elements (19.6% of all BES), simple sequence repeats and putative proteins, and to estimate the GC content (~42%) of the reads. Around 9.6% of all BES were found to have high levels of similarity to plant genes and ontological terms were assigned to more than half of the sequences analysed (940). The vast majority of the top-hits made by our sequences were to *Populus trichocarpa* (24.8% of the total occurrences), *Theobroma cacao* (21.6%), *Ricinus communis* (14.3%), *Vitis vinifera* (6.5%) and *Prunus persica* (3.8%).

**Conclusions:**

We generated the first large-insert library for a member of Passifloraceae. This BAC library provides a new resource for genetic and genomic studies, as well as it represents a valuable tool for future whole genome study. Remarkably, a number of BAC-end pair sequences could be mapped to intervals of the sequenced *Arabidopsis thaliana*, *V. vinifera* and *P. trichocarpa* chromosomes, and putative collinear microsyntenic regions were identified.

**Electronic supplementary material:**

The online version of this article (doi:10.1186/1471-2164-15-816) contains supplementary material, which is available to authorized users.

## Background

The family *Passifloraceae* belongs to the Order Malpighiales, one of the largest orders of the flowering plants and a member of the Clade Rosids according to classical and molecular phylogenetic analyses including several different gene sequences. The family consists of about 700 species of herbaceous or woody vines, shrubs, and trees, classified in around 16 genera, with almost all of its members belonging to the genus *Passiflora*, popularly known as passion fruits [1]. *Passifloraceae* is widely distributed in tropical and warm-temperate regions of the Neotropics and in Africa. Only a small number of species (~20) are found outside the Neotropics, in the Pacific and Indomalesian region. The hallmark of the family is the corona (or crown), more developed in insect-pollinated species. It stores the scent and nectar and also acts as a landing platform for pollinators.

The *Passiflora* species (~530) are typically tendril-bearing vines with a non-pedunculate inflorescence and one or two sessile, pentamerous flowers, many of which are highly prized for their exotic, unusual flowers. Three characters, the corona, operculum, and limen, have historically been used as taxonomic characters for delimiting relationships within *Passiflora* [2]. More recently, the genus was divided into four subgenera, *Astrophea* (57 species), *Decaloba* (220) *Deidamioides* (13), and *Passiflora* (240) [3]. In the subgenus *Astrophea* (*n* = *12*), shrubs or true trees tend to occur, distributed in lowland tropical South America. Species of *Decaloba* include herbaceous vines with small flowers and fruits occurring throughout the entire distribution of the genus and referred to as the ‘*n* = *6* group’. Lianas or herbaceous vines are typical in the *Deidamioides* subgenus (*n* = *12*), species of which are found throughout Central and South America and Mexico. One species occurs in New Zealand. Finally, the *Passiflora* subgenus comprises herbaceous or woody lianas with exotic flowers and edible fruits, distributed throughout Central and South America and the Southern US. The chromosome number led to the designation of this subgenus as ‘*n* = *9 group*’ [4].

The wide range of floral colours, sizes, and shapes is related to pollination (http://www.mobot.org/mobot/passifloraceae/). For instance, in subgenus *Astrophea*, large bee and hummingbird pollination is common, while bat pollination has been documented in the night-blooming passionflower (*P. penduliflora*), a representative of subgenus *Decaloba*. Interestingly, the genus has undergone changes in pollinators at various times. *P. roseorum* is an example of hummingbird pollination within subgenus *Passiflora*, in which bee pollination is predominant. Many new hybrids in subgenus *Passiflora* are created annually as growers try to develop novel ornamentals, since most species have conspicuous, scented, purple-banded coronas. Almost all the flowers bloom for less than a day.

There is substantial variation of genome sizes (1.0736 ± 0.56 pg) and flower diameters (6.126 ± 2.75 cm) in the genus [5]. The difference between the largest and smallest genomes was reported as high as 10 times (0.212 pg in *P. organensis*, *Decaloba*; 2.208 pg in *P. alata*, *Passiflora*). Both genome size and flower diameter means were smaller in *Decaloba* than in *Passiflora*, the two subgenera to which the 50 diploid (*n* = *9*) species investigated belong. Furthermore, a previous study has shown a wide genome size variation in a sample of eight taxa, ranging from 0.915 pg (*P. suberosa*, *n* = *12*) to 2.680 pg (*P. quadrangularis*, *n* = *9*) [6]. Note that most of *Passiflora* nucleotide sequences available from public databases are microsatellite sequences used to develop genetic markers [7, 8], transcription factors and internal transcribed spacers, ribosomal RNA genes, or partial gene sequences used in phylogenetic studies [9, 10]. In addition, information on genes involved in the flowering process of *Passiflora* spp. is accessible over the internet (passioma.ib.unicamp.br) [11].

As mentioned above, some *Passiflora* species are popular ornamentals, whereas others are grown for their edible fruits, including the giant granadilla (*P. quadrangularis*), the purple granadilla (*P. edulis*) and the yellow granadilla (*P. laurifolia*), all widely grown in Latin America. The purple passion fruit is also grown in Australia and Hawaii, and nowadays in Kenya, South Africa, Israel, and in North America. *P. maliformis* is the sweet calabash of the West Indies.

Commercial plantations of passionflowers are a somewhat recent phenomenon. In Brazil, the yellow passion fruit (*P. edulis* f. *flavicarpa*, *n* = *9*) is a crop of considerable economic importance both for juice production and consumption as fresh fruit. The plant is also used in the cosmetics and phytotherapeutic industries [12]. Brazil is the largest producer and consumer, with a cropping area of around 61,842 ha yielding some 923,035 tons a year [13]. Despite its commercial importance, very little is known about the genome of the passion fruit. Therefore, improving our understanding of passion fruit genome is very important and to some degree a pre-requisite if its genetic resources are to be used more efficiently.

Bacterial artificial chromosome (BAC) libraries have become an invaluable resource as a starting point for genomic research, and in particular genome sequencing, physical mapping, complex analysis of targeted genomic regions, and studies on gene structure and function [14–18]. Many important plant species are reported to have their entire genomes inserted in a BAC-based system [19–23], which remains the most commonly used large-insert cloning vector. Consequently, a very efficient and viable strategy for obtaining the initial insight into the content and complexity of a particular genome involves sequencing the terminal regions of a representative number of BAC clones selected at random from this genomic library. Such sequences, known as BAC-ends (BES), can be used for generating partial physical maps and performing comparative analyses [24]. BES data analysis provides an overview of genome composition, as well as information on gene density and the occurrence and distribution of transposable elements (TEs) and Simple Sequence Repeat (SSRs), essential data for the development of molecular markers [25–27].

In this study, we have constructed and characterized the first large-insert BAC library for a *Passiflora* species in order to facilitate genomic research. The long-term objective of our work is to study the structure, organization, and content of the yellow passion fruit genome, using BES data as a major resource. Specifically, our aims were (*i*) to construct a representative BAC library with six haploid genome equivalents using the estimation of 1,563 Mb genome size of *P. edulis* from Souza et al. [6]; (*ii*) to characterize the clones in relation to their insert sizes, chloroplast and mitochondrial DNA content; (iii) to demonstrate the utility of the BAC library in gene isolation and cytogenetic mapping; (*iv*) to use BES to sequence approximately 15% of the BACs; and (*v*) to compare our genome data with other data on related plant species. It is important to emphasize that our analysis, in terms of genome organization and sequence representation, is very preliminary and is mostly being used to assess the quality of the library.

## Results and discussion

### BAC library construction and characterization

The *Passiflora edulis* BAC library consists of 82,944 individual BAC stored clones in 216 (384-wells) plates and is kept at the French Plant Genomic Resource Center (CNRGV). The library has the following designation: Ped-B-Flav [http://cnrgv.toulouse.inra.fr/fr/library/genomic_resource/Ped-B-Flav].

**Figure 1:**
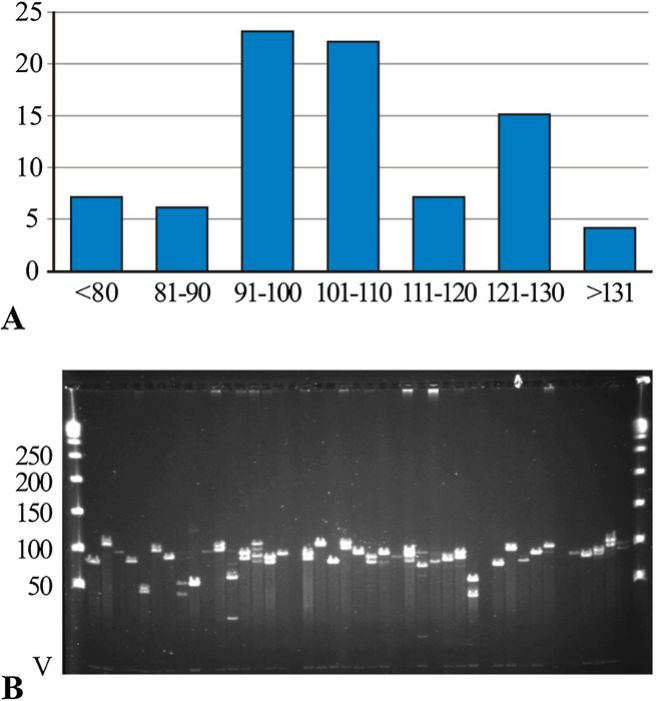
**Insert features of the BAC library. (A)** Insert size distribution in a random sample of clones from the library (percentages on the y-axis); most of the clones had an insert size larger than 90 kb. **(B)** A typical agarose pulsed-field gel showing *Not*I digests of clones. The Lambda Ladder PFG Marker (New England Biolabs) is in the lanes at far left and right. Marker bands are given in kb, and the size of the vector (V) is 7,506 bp.

To evaluate the medium insert size, 86 randomly chosen BACs were digested with *Not*I enzyme and analysed by pulsed field gel electrophoresis. The resulting restricted DNA typically showed the vector band and one or two insert bands per clone. The inserts ranged in size from 50 to 196 kb and the average insert size for the library was 108 kb. The insert size of most of the clones (87%) was larger than 90 kb (Figure 1). Our data confirmed that partial digestion of high molecular weight DNA with the restriction enzyme *Hind*III (1–2 U/plug as optimal concentration) was critical to produce fragments with a good size range, leading to a good quality of the BAC library containing fragments with acceptable insert sizes, important for future whole genome study.

**Figure 2:**
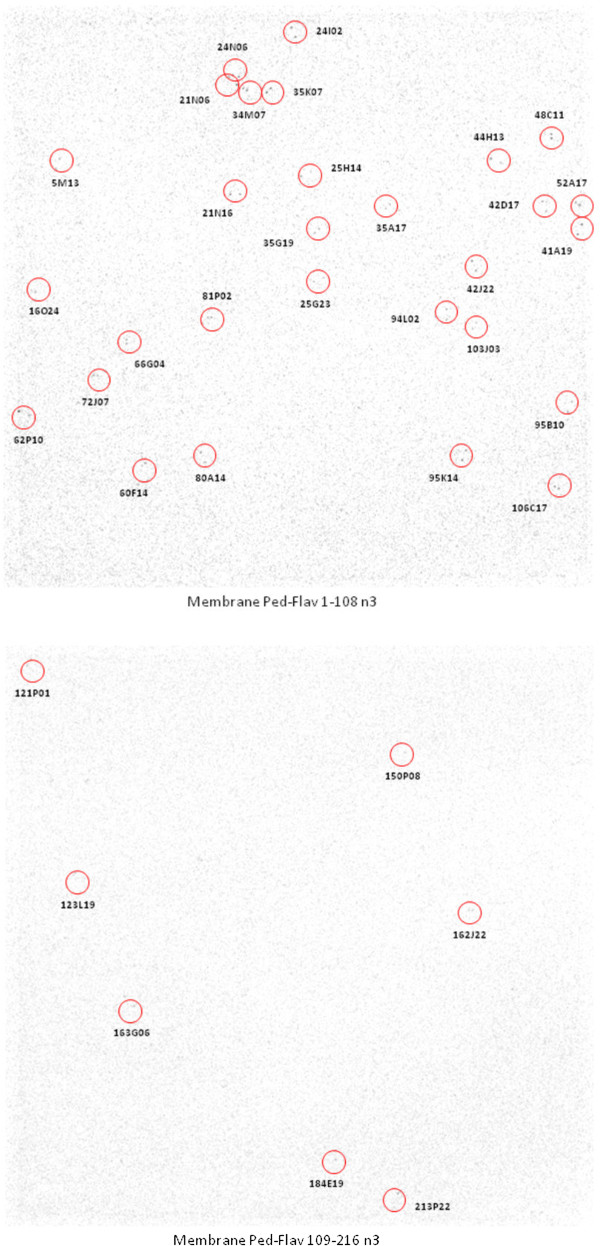
**Hybridization results of the** ***Passiflora edulis*** **BAC library screening using chloroplast gene probes,** ***psbA,*** ***psbB*** **and** ***ndhB.***

Additionally, the library has been gridded onto macroarrays (following a 6 × 6 pattern) in which 41,472 clones were double-spotted on each nylon membranes of 22 × 22 cm. Subsequently, the level of organellar DNA contamination was estimated by macroarray hybridization using radiolabeled mitochondrial and chloroplast gene probes. Hybridization results allowed the identification of 5 and 36 clones for mitochondrial and chloroplast genes, respectively (0.006% and 0.04%). Our results are consistent with the generally low frequency of mtDNA and cpDNA contamination observed in other plant BAC libraries such as in *Quercus robur* [27], *Camellia sinensis* [28] and *Pinus taeda* [29]. On the other hand, the identification of a number of BAC clones (36) containing cpDNA in the present study could provide a valuable basis for investigating the structure of the *Passiflora* chloroplast DNA (Figure 2).

To some extent, the above mentioned hybridization data confirmed the genome coverage of the library. Assuming that 41 clones contain organellar DNA, the number of clones harbouring yellow passion fruit nuclear DNA is 82,903. Since the haploid genome content of *P. edulis* is reported to be ~1.6 pg or 1,563 Mb [6], a library composed of 82,903 clones with an average insert size of 108 kb affords coverage of ~6 genome equivalents of nuclear DNA [(82,903 × 0.108 Mb) ÷ 1,563 Mb = 5.73]. The probability of isolating any single-copy DNA sequence from the *P. edulis* library would be greater than 99.7%, according to the Poisson distribution.

To check the utility of our library in gene isolation, macroarray screening experiments were carried out with radiolabeled probes complementary to eight *Passiflora* gene sequences available in public databases (Table 1). Hybridization allowed us to identify 78 positive clones. These clones were individually checked by PCR using the 8 paired primers of the genes chosen, and 18 of them (23%) corresponded to the 1-aminocyclopropane-1-carboxylate oxidase (*ACO1*) gene; 14 (18%), 10 (13%) and 5 clones (6.5%) corresponded to the sequences of glyceraldehyde-3-phosphate dehydrogenase (*G3PD*), lipoxygenase (*LOX*) and NADP-dependent isocitrate dehydrogenase (*NDID*) genes. Approximately 10% of the clones were specific of the primer pairs complementary to the ethylene response sensor (*ERS*, 9 clones) gene, embryo defective 2765 (*EMB*, 8) gene, cyclin-dependent protein kinase regulator (*CYCD1*, 7) gene and the myo-inositol 1-phosphate synthase (*MIPS*, 7) gene. All genes have a multifamily organization and therefore these differences are possibly due to random effects.

**Table 1:** ***Passiflora*** **genes, codes, and primer sequences used for screening the library**

Gene or gene product	Code	GeneBank accession no.	Forward (1 ^st^ line) and reverse (2 ^nd^ line) primer sequences	Predicted length (bp)
1-Aminocyclopropane-1-carboxylate oxidase	ACO1	AB015493	AGGATATGGACTGGGAGAGC	486
			TCGCACCATCTGTTTGAGC	
Embryo defective2765	EMB2765	FJ669803	TGAGAGTGTTGTGCCAAGC	523
			GAGGGTTCCTTCTTCATCAC	
Ethylene response sensor	ERS2	AB070652	GCTTATGCAGTTTGGTGC	647
			CAGAAAGATCAGGCCAATC	
Cyclin-dependent protein kinase regulator	CYCD1	HM003689	CTCTCCAGGTTTCAGTCTCG	587
			TTGCTTAGCCCATCACACC	
Glyceraldehyde-3-phosphate dehydrogenase	G3PD	AY858231	CATCCTTTCTGCGGTGATC	591
			CTTTCATGCTGCCCTCTG	
Lipoxygenase	LOX	GQ141712	AACATGCCAACCGAGGAC	482
			TTGATGGACATATCTGGAACC	
Myo-inositol 1-phosphate synthase	MIPS	DQ489558	AAATGAAGTCCGTCCTGGTG	565
			GGAGCCAATCCAATACAAGC	
NADP-dependent isocitrate dehydrogenase	NDID	AB304270	CAAGGTCGCTAATCCCATC	598
			TTTCTGGTAAGCCGTGTTC	

**Figure 3:**
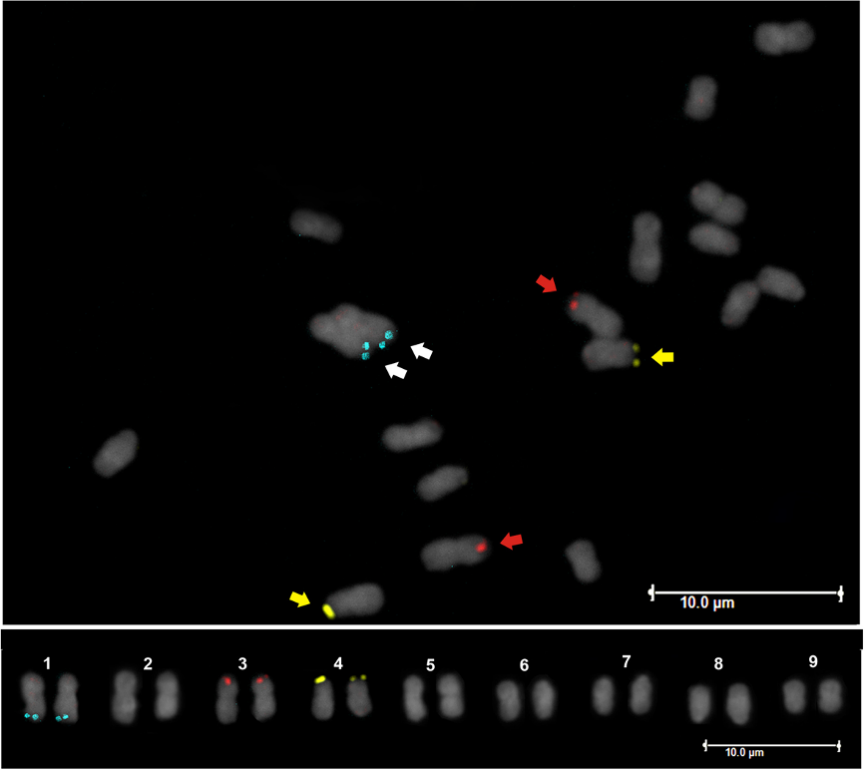
**Fluorescent** ***in situ*** **hybridization of** ***Passiflora edulis*** **mitotic chromosomes using BAC probes harbouring the following gene sequences:** ***ERS*** **(in blue),** ***ACO1*** **(in red) and** ***G3PD*** **(in yellow).** Slides were counterstained with DAPI. The corresponding karyogram (2*n* = 18) is shown in the bottom of the figure.

BACs harbouring those eight genes were used individually in fluorescent *in situ* hybridization (FISH) on somatic metaphase *P. edulis* chromosomes. Unique signals were detected for four BACs in three chromosomes of the complement (*n* = *9*). Using the *P. edulis* karyotype defined previously as reference [30–32], the sequence complementary to the *ERS* gene harboured by BAC198H23 was mapped to the long arm of putative chromosome 1, the *ACO1* gene sequence (BAC134H15) was mapped to the short arm of chromosome 3, and the *CYCD1* and *G3PD* gene sequences (BAC125I23 and 215I08, respectively) were both mapped to the short arm of chromosome 4, all in terminal positions (Figure 3). The remaining BACs resulted in strong and dispersed signals located preferentially in subtelomeric or pericentromeric regions of most chromosomes, indicating they are enriched in repeated DNA sequences. The library’s potential for building a *P. edulis* physical map based on the BAC-FISH approach was clearly demonstrated.

### BAC-end sequence analyses

In total, DNA from 5,974 BAC clones were purified for subsequent sequencing using an ABI 3500 × L automated platform. Of these BACs, 5,058 were sequenced at both ends (forward and reverse directions), and 916 (unpaired BACs) were sequenced in only one direction (512 forward and 404 reverse). Thus, 11,032 sequencing reactions were performed, yielding 9,698 good quality BAC-end sequences (or BESs), indicating efficiency of 87.39%. The average length of the sequences was 587 bp, ranging from the minimum acceptable size (100 bp) to 1,255 bp. The redundancy in the sequenced set was 5.4%. To summarize, our BAC-end sequencing effort generated approximately 6.2 Mb (6,194,248 bp) of genomic information for *P. edulis*, covering around 0.39% of its genome.

First, we searched in plant databases for mitochondrial and chloroplast sequences. Only 0.17% of the BAC-end sequences were similar to mitochondrial (2 BES) and chloroplast (15 BES) genome sequences. Next, the GC content of the passion fruit genome was estimated and found to be high (41.9%) compared to other dicots. Though preliminary, it is interesting to see that the value is high even when compared to the GC content of phylogenetically close species, such as *Vitis vinifera* (34.5%) [33], *Populus trichocarpa* (33.7%) [34] and *Ricinus communis* (32.5%) [35], and is close to the value usually found in monocots [36].

The GC content observed in the genic portion of the *P. edulis* genome was 45.0%, supporting previous findings which have shown that genes generally have a higher GC content in contrast to the background GC content of the entire genome. Similarly, we reported a GC content of 45.6% when examining an SSH library enriched for a set of sequences of *P. edulis* (‘IAPAR-123’) expressed in response to *Xanthomonas axonopodis* infection [37].

The average GC content of plant genomes varies enormously and reflects significant compositional features of the genomes. In dicots, for instance, the values generally range from 33% to 36% as reported for the genomes of *Carica papaya* (35%) [38], *Quercus robur* (35.3%) [27], and *Arabidopsis* (36%) [39]. Monocots tend to have higher GC contents, generally between 43% and 47%. The highest values are found in grasses, which may be explained by the proliferation of GC-rich retrotransposons and the presence of grass genes that are extremely GC-rich.

### Prospecting for retro- and transposable elements and microsatellite sequences

Sequences from Repbase were used to delineate the occurrence of different classes of repetitive elements within the *P. edulis* genome, screening out the fraction that corresponds only to sequences of *Viridiplantae*. Repetitive elements were recognized in 19.6% of all BES (Table 2), covering approximately 1.12 Mb (or 1,215,879 masked nucleotides). Compared to other studies that used BES to provide an overview of the composition of plant genomes, the inference of the amount of repetitive elements in *P. edulis* is reasonable. Lower values were found in *Quercus robur* (~5.9%) [27], *Cucumis melo* (6.0%) [40] and *Juglans regia* (15.4%) [41] but much higher estimates were made for dicots, *Citrus clementina* (25.3%) [42] and *Daucus carota* (28.3%) [26], and in the monocots *Phalaenopsis equestris* (23.0%) [43] and *Musa acuminata* (36.6%) [44]. Evidently, part of this variation can be explained by technical factors, i.e. the quality and quantity of sequences analysed.

**Table 2:** **Classification and distribution of the repetitive elements detected among** ***Passiflora edulis*** **BAC-end sequences**

Class	Number of elements	Number of masked nucleotides	Percentage of nucleotides
**Class I retroelements**	**3,** **401**	**1,** **144,** **329**	**18.47**
*LTR elements*	*3*,*327*	*1*,*123*,*137*	*18.13*
Ty1/Copia	1,606	487,318	7.87
Gypsy/DIRS1	1,706	630,816	10.18
Unclassified LTRs	15	5,003	0.08
*LINEs*/*SINEs*	*74*	*21*,*192*	*0.34*
L1/CIN4	71	21,043	0.34
Other LINEs/SINEs	3	149	0.00
**Class II DNA transposons**	**38**	**2,** **881**	**0.05**
hobo-Activator	7	644	0.01
Tourist/Harbinger	1	47	0.00
Mirage	1	37	0.00
Unclassified transposons	29	2,155	0.04
**Unclassified elements**	**7**	**1**,**192**	**0.02**
**Small RNA**	**2**	**162**	**0.00**
**Satellites**	**1**	**41**	**0.00**
**Simple repeats**	**327**	**15,** **514**	**0.25**
**Low complexity**	**1,** **000**	**51,** **760**	**0.84**
**Total repetitive DNA**	**4**,**776**	**1,** **215,** **879**	**19.63**

Total number of nucleotides examined: 6, 194, 248.

On the other hand, compared to estimates reported for other dicot genomes already sequenced, including the more closely-related species *Populus trichocarpa* (42.0% of ~480 Mb) [34] and *Vitis vinifera* (41.4% of 416 Mb) [33] as well as *Malus* × *domestica* (42.4% of 881 Mb) [45] and *Carica papaya* (~52% of 372 Mb) [46], we found a much lower percentage of repetitive elements in *P. edulis*.

The elements annotated as Class I were significantly more abundant than Class II (Table 2). There is a robust consensus in the literature, that the larger the plant genome, the greater the chance it contains many retrotransposons. For example, the large genome of barley (5,428 Mb) comprises up to 70% retrotransposons [47], while in the small rice genome (489 Mb) these elements represent only 17% of its total composition [48]. In the present study, retrotransposons were identified in about 18.5% of all BES. Proportionally, this value is not high, but we need to take into account the fact that *P. edulis* has a larger genome on average, than most dicots. Quantitatively, that is to say 18.5% of a genome that can reach 1,563 Mb is a large amount of the transposable elements.

Long terminal repeats (LTRs), such as Ty1-copia and Ty3-gypsy retrotransposons, are enriched in pericentromeric regions, playing important roles in maintaining chromatin structures and centromere functions and regulating gene expression in the host genomes [49]. A parameter commonly used in BAC-end analyses is the proportion of LTR retrotransposons Ty3-gypsy relative to Ty1-copia. These groups differ from each other in both their degree of sequence similarity and the order of encoding genes. We observed an approximate ratio of 1:1, indicating that the contributions of Ty3-gypsy and Ty1-copia to the *P. edulis* genomes were apparently equal (Table 2). Very distinct proportions were reported to occur in plants: 1:2 in *Quercus robur* [27], 1:3 in both *Malus* × *domestica* [25] and *Vitis vinifera* [33], and 2:3 in *Saccharum* spp. [50] where Ty1-copia outnumbered Ty3-gypsy transposons. Ratios of 3:1 and 5:1 were found in *Oryza sativa* [51] and *Phalaenopsis equestris* [43], respectively.

In the present study, Non-LTR elements included 74 (0.34% of all repetitive elements identified) belonging to the LINE/SINE category. LINEs are sequences (up to 7,000 bp long) able to transpose autonomously, while SINEs (75–500 bp long) depend on the reverse transcription machinery of other retrotransposons and both are of great importance in the evolutionary history of plant genomes. Class II DNA transposons were found infrequently (0.34%) as were the remaining repetitive elements (simple repeats and elements of low complexity) consisting in approximately 1% of all repetitive elements. Similar rates were reported among walnut (*Juglans regia*) BAC-end sequences [41].

It is worth noting that species of *Populus* are predominantly dioecious, and therefore outcrossing and wind pollinated, resulting in high levels of gene flow and heterozygosity [34, 52]. Similarly, *P. edulis* is an obligate outcrossing species based on self-incompatibility and insect-pollination. However, there is evidence of low levels of allelic polymorphism or heterozygosity [7], as already documented in *Passiflora alata*, the sweet passion fruit [53]. Speculating about *Populus trichocarpa* has a small genome (~480 Mb) [34] and *Passiflora edulis* a large genome (1,563 Mb) [6], but the *P. edulis* genome seems not to accumulate repetitive elements in contrast to *P. trichocarpa* which is described as a large repetitive genome.

Before masking repetitive regions and transposable elements (Class I and II), sequences were subjected to a search for microsatellites or Simple Sequence Repeat (SSRs). After running the automatic search, each chromatogram was manually checked to ascertain the quality and reliability of automatically annotated peaks. In total, 669 microsatellite sequences were found in the 6,194,248 bases analysed, an average of 10.8 SSRs every 100 kb or one SSR every 9.25 kb. Our estimate is close to the figures documented for other plants, such as *Brassica rapa* (10.46) [54] and *Solanum tuberosum* (11.57) [55]. However, it is low in comparison to the average density of microsatellites in two phylogenetically close species, *V. vinifera* (17.24) [33] and *P. trichocarpa* (18.89) [34].

**Figure 4:**
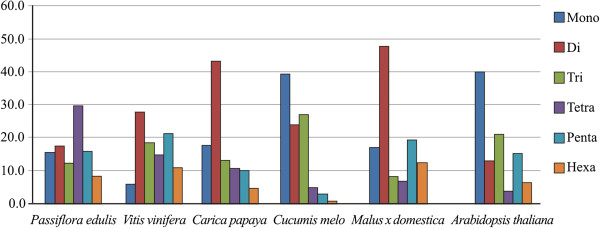
**Comparison of the percentage of simple sequence repeats (SSRs) detected in BAC-end sequences of** ***Passiflora edulis*** **(9,751 BES),** ***Vitis vinifera*** **(31,907),** ***Carica papaya*** **(6,270),** ***Cucumis melo*** **(23,878),** ***Malus*** **×** ***domestica*** **(3,744) and** ***Arabidopsis thaliana*** **(26,068), according to the repeat motif (mono-, di-, tri-, tetra-, penta-, and hexanucleotide).**

SSRs were grouped according to motif, and we were able to identify all possible classes of repeats. Altogether, 15.7% of the SSRs were mono-, 17.7% di-, 12.5% tri-, 29.8% tetra-, and 15.9% pentanucleotides. Only 8.4% of the SSRs were hexanucleotides. For comparison purposes, analyses were performed to identify microsatellites in data sets from BAC-end sequences of other fruit species, *Vitis vinifera*, *Carica papaya*, *Cucumis melo*, *Malus* × *domestica*, as well as *Arabidopsis thaliana*. These public data were obtained from the NCBI website (http://www.ncbi.nlm.nih.gov) using the same software and parameters used in the exploitation of microsatellites in BAC-end sequences of *P. edulis*. The results of these comparisons are shown in Figure 4.

Among the mononucleotides, A/T exceeded by far the proportion of G/C repeats (62% compared to 38%). In fact, analyses carried out on *Arabidopsis thaliana*, *Vitis vinifera* and *Populus trichocarpa* have shown that, in general, dicots have a high frequency of A/T-rich microsatellites. The most frequent dinucleotides were AT, followed by AG repeats. None of the classes contained significant frequencies of GC-rich motifs. This is consistent with studies on *Arabidopsis thaliana* [56], *Carica papaya* [38], and *Malus* × *domestica* [25] showing that AT-rich sequences are much more prevalent in the genomes of higher plants. In *P. edulis*, the motif distribution within repeat classes follows the same tendency observed in other plant BAC-end sequences. Finally, the most abundant motif was the tetranucleotide AATT (149 occurrences).

Interestingly there is a high incidence of tetranucleotide motifs (~30%) compared to other classes of microsatellites in our data set. This arrangement is unusual in dicots, where dinucleotides or mononucleotides are normally the most abundant repeats. Exploiting a microsatellite-enriched library of *P. edulis*, skewed for dinucleotides due to the probes used in its preparation, Oliveira and co-authors also observed a higher proportion of tetranucleotides compared to other microsatellite classes [7].

### Protein coding regions

**Figure 5:**
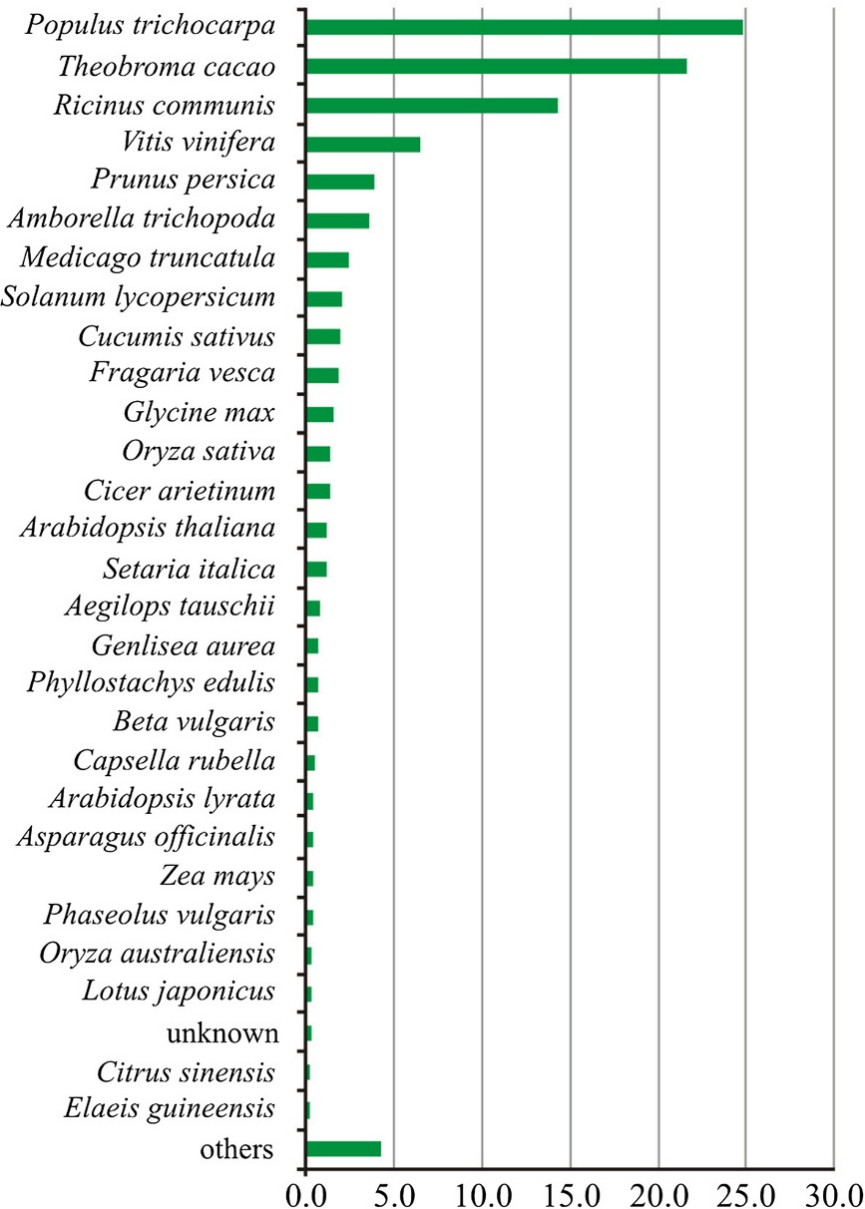
**List of species that produced significant hits with the BAC-end sequences of** ***Passiflora edulis*** **(940) based upon sequence similarity to plant genes (best hit rate at the top).**

With the aim of identifying protein-coding regions, the BAC-end sequences were compared to the non-redundant protein data bank using the BlastX search engine (http://www.ncbi.nlm.nih.gov/BLAST). Around 9.6% of all BES (940 sequences) were found to have high levels of similarity (e-values smaller than 1 × 10^−6^) to genes of several plant species. The vast majority of the top-hits made by our sequences were to *Populus trichocarpa* (24.8% of the total occurrences), *Theobroma cacao* (21.6%), *Ricinus communis* (14.3%), *Vitis vinifera* (6.5%) and *Prunus persica* (3.8%). This scenario was expected since these are species related to *P. edulis* on which there is an abundance of genomic data. The list of species hits in descending order is shown in Figure 5.

Assuming that 9.6% of the BES analysed had matching coding regions, we can estimate that ~151 Mbp of the passion fruit genome consist of coding sequences. Assuming an average gene length of 3.4 kb, the same as *Vitis vinifera* [33], we can estimate that the total number of genes per haploid genome of *P. edulis* is 44,000. Both the estimate of the coding portion and the number of genes are consistent with the figures proposed for *Phaleanopsis equestris*, a species with a genome size very close to that of *P. edulis* [43]. Our approximation is within the range of the gene numbers estimated for the closest relatives *Vitis vinifera* (30,434 genes) [33] and *Populus trichocarpa* (45,555 genes) [34].

Altogether 2,688 ontological terms were assigned to 507 of the 940 sequences analysed, resulting in an average of 5.3 terms per sequence. Up to 32 GO terms were annotated for the sequences, and those related to molecular function were the most abundant. Of the total sequences with assigned ontological terms, 409 were assigned to at least one term related to molecular function, 403 to at least one term related to biological process, and 299 to at least one term related to cellular component. Any term was assigned to 248 of the sequences which contained matches with coding sequences, and 185 of the sequences were mapped but not annotated.

**Figure 6:**
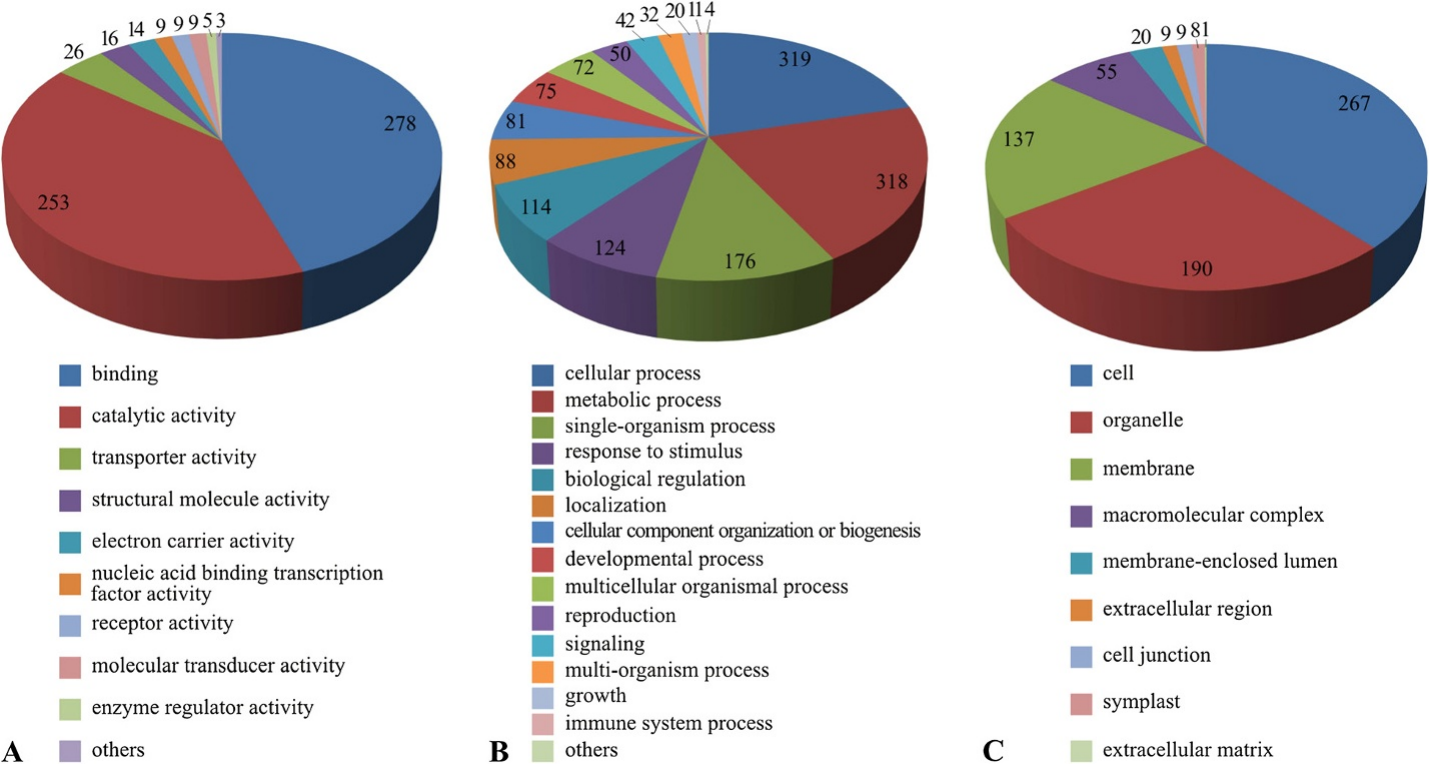
**Distribution of GO annotations of gene products predicted from the** ***Passiflora edulis*** **BESs for molecular function (A), biological process (B) and cellular component (C).** Only major categories are presented.

Within the category molecular function, the most frequent terms were binding (GO: 0005488), catalytic activity (GO: 0003824) and transporter activity (GO: 0005215) (54.8%, 49.9%, and 5.1% of occurrences, respectively). Within the category biological process, the most frequent terms were cellular process (GO: 0009987), metabolic process (GO: 0008152), single-organism process (GO: 0044699), and response to stimulus (GO: 0050896) (62.9%, 62.7%, 34.7%, and 24.4% of occurrences, respectively). Finally, in the category cellular component, the most common terms were cell (GO: 0005623), organelle (GO: 0043226) and membrane (GO: 0016020) (52.7%, 37.5% and 27.0% of occurrences, respectively) (Figure 6).

### Comparative genome mapping

**Figure 7:**
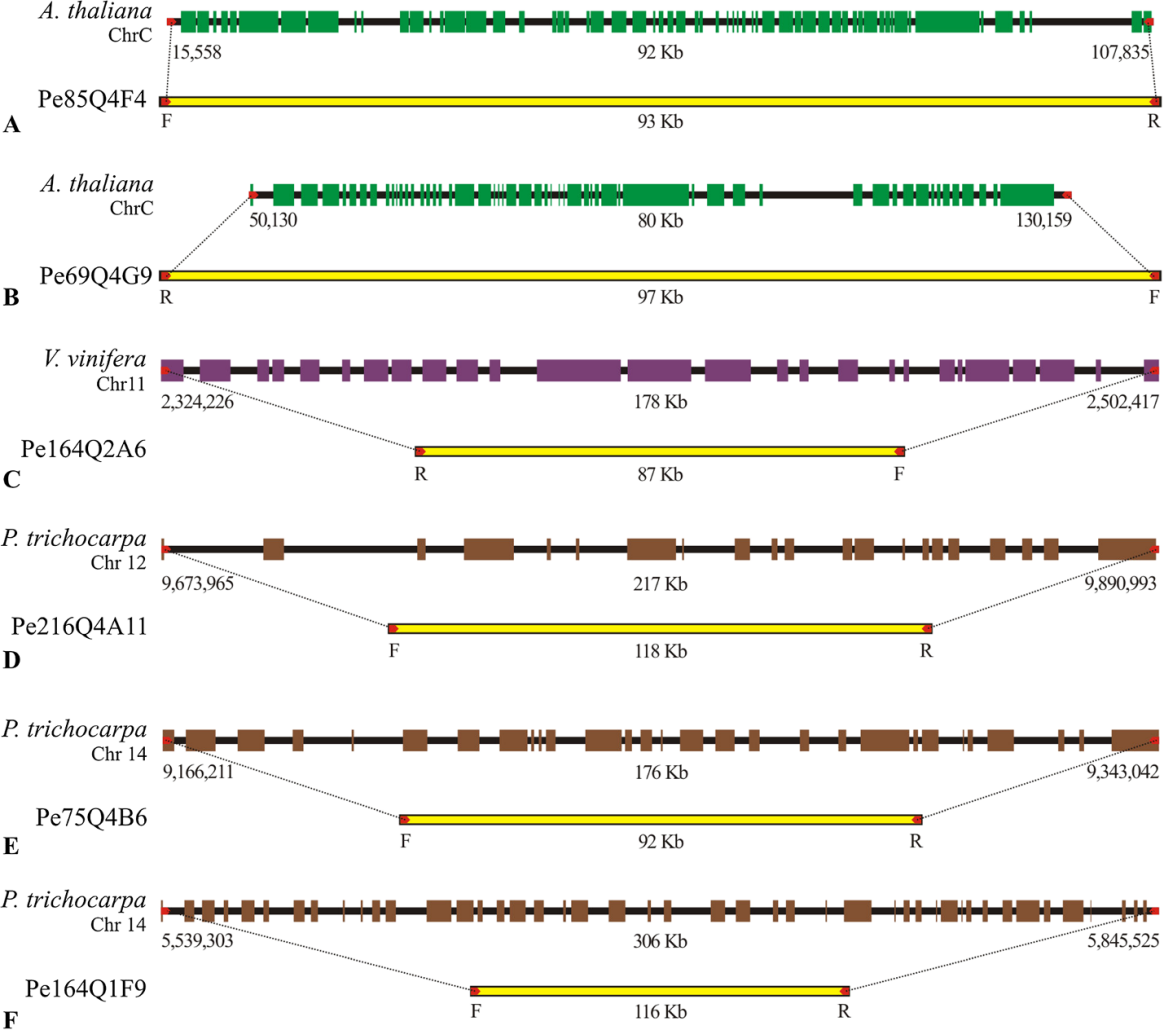
**Comparison of collinear microsyntenic regions identified in** ***Passiflora edulis*** **BACs (yellow bars showing the respective paired ends, F and R) and** ***Arabidopsis thaliana*** **(A, B),** ***Vitis vinifera*** **(C) and** ***Populus trichocarpa*** **(D, E, F) chromosomes.**

In total, 9,698 BES were compared to the sequenced *Arabidopsis thaliana*, *Vitis vinifera*, and *Populus trichocarpa* genomes for identifying microsyntenic regions (Figure 7, Additional file 1: Table S1). *P. trichocarpa* and *V. vinifera* are among the plant genomes with the highest number of top hits for *P. edulis* (Figure 5). Although phylogenetically closer, the *Ricinus communis* and *Prunus persica* genomes are still in the early stages of their assembly and annotation, which enormously affects comparative mapping studies. The same is true for the draft genome of *Theobroma cacao*. For obvious reasons, *A. thaliana* has been chosen as a default template.

The matches (any occurrence of similarity between a *P. edulis* BES and a specific region used for comparing genomes) were classified into six categories based on the classification in Rampant and co-authors [27]. The ‘single end’ (SE) category refers to BAC end pairs for which only one of the ends (F or R) matched a sequence in the reference genome; BES were assigned to the ‘paired end’ (PE) category when both end sequences matched the genome. The PE category, in turn, is subdivided into ‘co-localized’ (BAC end pairs that matched the same reference chromosome) and ‘non co-localized’. We considered all matches occurred at points 15 to 350 kb apart on the reference genome; either in the correct orientation with respect to each other (‘collinear’) or rearranged with respect to each other (‘rearranged’). When the distance between the paired matches was either smaller or larger than these limits, they were assigned to the ‘gapped’ category.

First, we identified 106 positive matches to *A. thaliana* genome, 100 SEs and 6 PEs. Four PEs formed two ‘collinear’ alignments, composed of sequences Pe85Q4F4 (F/R, 93 kb) and Pe69Q4G9 (F/R, 97 kb), encompassing 58 and 55 genes, respectively. The lengths of potential microsyntenic regions found in *A. thaliana* are close to the average insert sizes of the *P. edulis* BAC library. For instance, the *Arabidopsis* region shared at its ends by the Pe85Q4F4 sequences is 92 kb in length; the respective *P. edulis* BAC is 93 kb. Similarly, the *Arabidopsis* region shared by the Pe69Q4G9 sequences is 80 kb in length; the respective *P. edulis* BAC is 97 kb in length. It is interesting to note that *Arabidopsis* regions overlap each other covering a region of 114.6 kb, from chromosome C (Chloroplast) positions 15,558 bp (Pe85Q4F4F) to 130,159 bp (Pe69Q4G9F). Clearly, this kind of information can be applied to assemble the two *P. edulis* BACs that harbour the mentioned sequences. In addition, the pair Pe164Q1G7 (F/R, 97 kb) was classified as ‘rearranged’.

It is noteworthy that the ends of the PE Pe85Q4F4 showed sequence similarity to a 30S subunit ribosomal RNA (e-value = 1.2e^−27^) and to a membrane hydrolase (e-value = 8e^−36^). Additionally, the ends of the Pe69Q4G9 PE showed sequence similarity to an unspecified subunit of the NADH complex (e-value = 9.1e^−17^) and to a portion of subunit 3 of the NADH complex (e-value = 1.9e^−35^). According to cellular component annotation analysis (level 2), the four sequences belong to ‘chloroplast’ group. We are possibly facing a cpDNA contamination not detected on the macroarrays.

Comparing genome intervals shared by *P. edulis* and *V. vinifera*, we identifed 206 positive matches, 196 SEs and 10 PEs. Among the PEs, there were two ‘non co-localized’ pairs (Pe85Q4F4, F/R, 93 kb and Pe214Q2A9, F/R, 109 kb), one ‘collinear’ pair (Pe164Q2A6, F/R, 87 kb), one ‘rearranged’ pair (Pe164Q1G7, F/R, 97 kb), and one ‘gapped’ pair (Pe69Q4G9, F/R, 97 kb). The paired sequences Pe164Q2A6, F/R were mapped to chromosome 11 of *V. vinifera*, the forward end beginning at position 2,502,417 bp of the grapevine genome and the reverse end terminating at position 2,324,226 bp, separated by around 178.2 kb. This region encompasses 26 genes, including small genes consisting of single exons, and large genes consisting of more than 10 exons. Compared to the *P. edulis* interval, the region in question is twice the length of the BAC Pe164Q2A6 (87 kb).

Finally, comparative mapping between the *P. edulis* and *P. trichocarpa* genomes revealed a large number of microsyntenic regions. Altogether, positive matches were found in 367 *P. edulis* sequences, including 333 SEs and 34 PEs. Three paired sequences of *P. edulis* were ‘collinear’ to those of *P. trichocarpa*. In addition, Pe173Q4A8 (F/R, 112 kb) was classified as ‘rearranged’ and Pe164Q1G7 (F/R, 97 kb) as ‘gapped’. The first ‘collinear’ pair Pe216Q4A11 (F/R, 118 kb) is located on chromosome 12 of *P. trichocarpa*, over an interval consisting of ~217 kb of DNA harbouring 21 genes. The second pair, Pe75Q4B6 (F/R, 92 kb) is separated by ~176 kb, including around 29 genes, and the third, Pe164Q1F9 (F/R, 116 kb) is separated by a 306 kb interval in *P. trichocarpa* genome that contains 45 genes involved in a number of different physiological processes. All these paired sequences were correctly orientated and separated by larger intervals in the *P. trichocarpa* genome, compared to *P. edulis*. It is interesting to note that paired sequences Pe75Q4B6 and Pe164Q1F9 were both assigned to chromosome 14 in *P. trichocarpa*, making these BACs good candidates for a BAC-by-BAC sequencing project based on a BES database.

The pair consisting of Pe173Q4A8 (F/R, 112 kb) sequences was classified as ‘rearranged’ because of their incorrect orientation with respect to each other, and the pair consisting of Pe164Q1G7 (F/R, 97 kb) sequences was classified as ‘gapped’, since the distance between the sequences is around 6 Mb, much larger than the maximum acceptable span. This huge distance could be the result of a major chromosome event in an ancestral genome, for instance, involving massive insertion as the predominant event. It is interesting that this pair was also mapped in the genomes of *A. thaliana* and *V. vinifera*, but spanning much smaller intervals.

The PEs included 24 classified as ‘non co-localized’ (on different chromosomes). These events are possibly due to macro chromosomal rearrangements that occurred since the split of *P. trichocarpa* [34], a hypothesis that finds support in the fact that *P. trichocarpa* (*n* = *19*) has twice as many chromosomes as *P. edulis* (*n* = *9*).

The above results are summarized in Additional file 1: Table S1. We expected to find a number of potentially microsyntenic regions between the *P. edulis* and *P. trichocarpa* genomes since the latter is evolutionarily closer evolutionarily to *Passiflora* than *V. vinifera* and *A. thaliana*. Obviously ‘collinear’ relationships tend to erode as we move further away from the genus *Passiflora*.

## Conclusions

We explored approximately 10,000 BAC-end sequences (BES) of the yellow passion fruit (*Passiflora edulis*) genome after generating a BAC library for the species. This is the first large-insert library for a member of Passifloraceae and a valuable tool for future sequencing and genome studies. We were able to verify the quality of the library (approximately 83,000 clones with very low mtDNA and cpDNA contamination), its genome coverage (~6 ×) and utility for gene isolation. This was done using exclusively the few *Passiflora* gene sequences deposited in public databases as probes in macroarray hybridizations, and the corresponding BACs used in FISH experiments allowed us to map four genes to three chromosomes of the species. We were able to identify reads likely to contain repetitive mobile elements (19.6% of all BES), simple sequence repeats and putative proteins. We estimate a ~42% GC content (~42%) from the analysis of the reads. Approximately 900 BAC end-sequences contained protein sequences, and ontological terms were assigned to 507 of them. Comparison analyses of the sequences generated were performed against sequence data from two phylogenetically close species *P. trichocarpa* and *V. vinifera*, as well as from *A. thaliana*. Around 680 positive matches or sequence similarities were detected. Finally, a number of BAC-end pair sequences could be mapped to intervals of *A. thaliana*, *V. vinifera* and *P. trichocarpa* chromosomes, representing regions of potential microsynteny.

## Methods

### Plant material

The genotype ‘IAPAR-123’ of *P. edulis* f. *flavicarpa* (*n* = *9*) was selected for BAC library construction since it was used as the female parent of a singlewide cross to generate a dense genetic map for the species [7]. It displays favorable characteristics with respect to total soluble solids, acidity, fruit yield and juice yield. Plant clones were grown in the dark for about three days. Young leaves were collected, rapidly washed in double-distilled water, and immediately frozen by submersion in liquid Nitrogen, followed by a short-term storage (−80°C).

### BAC library construction

The BAC library was constructed at CNRGV [57]. More specifically, 30 g of frozen tissue was grounded to a powder with liquid N. HMW DNA was extracted following the protocol described by Peterson et al. [58] and modified as described in Gonthier et al. [59].

Three DNA plugs were used to establish optimal partial digestion conditions, 1, 1.4 and 2 units of *Hind*III restriction enzyme added to each sample. The DNA separation was performed in two rounds. First, partially digested DNA was put into the wells of a 1% low-melting temperature agarose and separated by pulsed-field gel electrophoresis (PFGE CHEF-DRIII apparatus, Biorad, 6 V/cm, 50 s switch time, 18 h run time, 12.5°C). The region containing DNA fragments ranging in size from 80 to 250 Kb was excised from the unstained gel and divided into three equal slices that were embedded into a second gel and separated by PFGE (6 V/cm, 3–5 s switch time, 16 h run time, 12.5°C). After finishing the second selection, gel fractions were recovered and electro eluted from the agarose at 4°C. After estimating DNA concentrations, 120–200 ng were ligated to pIndigoBAC-5 vector and incubated under temperature-cycling conditions. Two μl of the ligation product was used to transform 18 μl of DH10B electro competent cells (Invitrogen) by electroporation at 13 KV/cm. One ml from the transformation mixture was spread on Q-trays (22.5 × 22.5 cm) with LB-agar medium containing X-gal (20 mg/ml), IPTG (0.1 M) and chloramphenicol (12.5 μg/ml), and incubated at 37°C overnight. A total of 82,994 recombinant clones were robotically picked (QPix2 XT, Genetix) and arrayed into 384-well plates (216 plates in total) containing sterile freezing medium, and stored at −80°C.

### BAC-insert sizing and screening the library for organellar DNA sequences and some *Passiflora*genes

The average insert size was assayed isolating 86 recombinant clones that were grown at 37°C overnight in 1.2 ml LB containing X-gal, IPTG and chloramphenicol with shaking (225 rpm). Samples (30 μl) of DNAs were isolated using a BAC DNA purification kit (Large-Construct Kit, QIAGEN), digested with 5U of *Not*I enzyme and size-fractioned by PFGE (6 V/cm, 5 up to 15 s switch time, 16 h run time, 12.5°C) followed by ethidium bromide staining and visualization. The size of the insert in each BAC clone was determined by comparison with PFGE standard size markers (New England Biolabs).

The BAC clones were gridded onto 22 × 22 cm Immobilon-Ny^+^ membrane (Millipore) using a robot (QPix2 XT, Genetix), following a 6 × 6 pattern. This gridding pattern allowed 41,472 colonies to be double spotted on each filter. Colony filters were incubated (17 h, 37°C) on solid LB medium plus chloramphenicol plated onto Q-trays, followed by transference to 4°C just until colonies became confluent. Membranes were kept (4 min) onto a Whatmann 3 MM paper saturated with denaturation buffer (0.5 M NaOH, 1.5 M NaCl), treated with the same buffer (10 min, 100°C), and neutralized (10 min) on Whatmann 3 MM paper saturated with neutralization buffer (1.5 M Tris–HCl pH 7.4, 1.5 M NaCl). Immediately, membranes were incubated (45 min, 37°C) with 250 mg/l proteinase K in 100 mMTris-HCl pH 8, 50 mM EDTA, 0.5 M NaCl. Finally, membranes were dried (45 min, 80°C) and UV-crosslinked (120,000 μJ/cm, 50 s).

To estimate the level of contamination by organellar DNA, membranes were hybridized with probes to target three mitochondrial (*ccb256*, *ccb452* and *cox3*) and three chloroplast (*psbA*, *psbB* and *ndhb*) genes. Probes were generated by PCR, using the DNA from the same passion fruit accession as template and specific primers. PCR products were purified with the NucleoSpin Extract II kit (Macherey-Nagel). The probes were labeled with [^32^P]dCTP the using Ready-To-Go DNA Labelling Beads (−dCTP) kit (GE Healthcare), and unincorporated nucleotides were then removed using ProbeQuant^TM^ G-50 Micro Columns G-50 (GE Healthcare). A set of probes was also used to facilitate the detection of hybridization to eight *Passiflora* gene target sequences deposited in Genbank. These genes were the only ones whose sequences were publicly available; details about gene codes and products, and primer sequences used for screening the library are shown in Table 1.

### Fluorescent *in situ*hybridization

Root tips obtained from germinating seeds of the passion fruit accession ‘IAPAR-123’ were pre-treated in 2 mM 8-hydroxyquinoline for 5 h at 18°C, fixed in ethanol-acetic acid 3:1 (v/v) and stored in fixative (−20°C). Mitotic chromosome preparation and selection were performed as previously described [60].

All BAC clones that showed positive signals after library screening (see Table 1) were spotted onto an N + Hybond membrane (Roche) as described above and hybridized with genomic DNA to [61]. DNA from BACs showing the least amount of repetitive DNA for each gene was extracted using the QIAGEN Plasmid Mini Kit. Probes were labeled by nick translation (Roche) with Cy3-dUTP or Cy5-dUTP (GE Healthcare).

The FISH procedure was essentially the same as previously described [62]. Hybridization mixes consisted of 50% (v/v) formamide, 10% (w/v) dextran sulfate, 2× SSC, and 2–5 ng/μl probe. The *P. edulis* C_0_*t*-100 fraction, isolated according to Zwick and co-authors [63], was added in 50- or 100-fold excess to the hybridization mix to block repetitive sequences when necessary. The slides were denatured for 5 min at 75°C in the hybridization mix and hybridized for up to 2 days at 37°C. The final stringency was 76%. Preparations were counterstained and mounted with 2 μg/ml DAPI in Vectashield (Vector). Re-probing of slides for localization of different DNA sequences on the same cell was performed according to Heslop-Harrison and co-authors [64]. The best slides were analysed using a Leica DMLB microscope and images were captured with a Cohu digital camera using the QFISH software (Leica). Images were superimposed and artificially colored using the Adobe Photoshop software version 10.0 (Adobe Systems) and adjusted for brightness and contrast only.

### DNA extraction for BAC-end sequencing

Bacterial clones were inoculated in 96-well plates (0.8 ml) containing 200 μl of 2× LB medium plus 1.25 μg/ml chloramphenicol. These, in turn, were dipped in 384-well plates and left momentarily in contact with the culture medium. The plates were then incubated (37°C) under constant shaking (325 rpm) for 7 h. A new subculture was prepared in 96-well plates (0.8 ml) containing 1.5 ml of 2× LB medium plus chloramphenicol. Sequentially, these plates were incubated at (37 ° C, 325 rpm) for a minimum period of 18 h. Next, the plates were centrifuged, (2,500 *g*, 10 min), the supernatant discarded and pellets stored (−20°C).

All minipreps were performed using NucleoSpin® 96 Flash kits (Macherey-Nagel®). The manufacturer’s protocols were modified so that the kits could be used efficiently on a large-scale. After thawing, each pellet was resuspended with the addition of 300 μl of a resuspension solution plus RNase, followed by a stirring period (650 rpm, 30 min). Then, 300 μl of a lysis solution were added and plates reversed (10 to 15 times), letting the solution sit for 4 min. Next, 300 μl of neutralization solution were added, followed by 15 reversal movements. Solutions were ultra-filtered (NucleoVac 96) and centrifuged (2,500 *g*, 1 h) after adding 630 μl of isopropanol (0.7 volumes). The supernatant was then discarded, 500 μl of 70% ethanol added, and a new centrifugation performed (2,500 *g*, 30 min). Finally, the supernatant was discarded and pellets allowed to dry (37°C for 1 h). Each miniprep was resuspended in 35 μl of ultrapure water and left to stand (4°C for 24 h) for subsequent freezing.

Reactions were performed using Sanger’s sequencing method, the BigDye® Terminator v3.1 Cycle Sequencing kit (Applied Biosystems), and the following universal primers: T7-Forward (5′ TAATACGACTCACTATAGGG 3′) and M13r-Reverse (5′ CAGGAAACAGCTATGAC 3′). The manufacturer’s reaction conditions were modified to improve cost effectiveness and ensure sequence quality with minimum size. Each reaction contained 2000–2500 ng of plasmid DNA, 2 μl of 5× Big Dye buffer®, 1 μl of dye, 0.2 μl of primers (50 mM), adding ultrapure water for a final volume of 15 μl. Amplifications were carried out using the following thermal cycling conditions: initial denaturation at 95°C (5 min) followed by 99 cycles of 95°C for 30 s, 50°C for 15 s and 60°C for 4 min. Reaction products were precipitated by adding 2.5 μl sodium-acetate (1.5 M), 2.5 μl of EDTA (250 mM) and 35 μl of cold absolute ethanol. Each pellet was resuspended in 10 μl of formamide. Sample injection and capillary electrophoresis were performed on an ABI 3500 xL DNA analyzer (Applied Biosystems). PHRED software [65] was used for base calling and assignment of base quality values, and vector and low quality-sequences were removed from the reads on the Egene platform [66].

### Identification of transposable elements and microsatellite sequences

The repetitive elements present in BES were identified by searches for similarity to sequences in the Viridiplantae section of the RepBase repeat database (released April 18, 2012), using the RepeatMasker ver 3.3.0 and Cross Match ver 0.990329 search engines and a cutoff value of 1 × 10^−20^. The density of repetitive elements was calculated as the percentage of nucleotides in the BES with at least one hit matching the repeat database. Repetitive element families were classified based on the annotation in the RepBase database, following the guidelines for classification and identification in the TIGR Codes for Repetitive Plant Sequences [67]. Once identified, the repetitive element regions were masked to avoid bias in further analysis.

BES-containing microsatellites were identified using SciRoKo ver 3.4 [68]. Microsatellites with mononucleotides spanning at least 10 nucleotides were recorded, as well as dinucleotides spanning at least five repeats, and tri-, tetra-, penta and hexanucleotides spanning at least three repeats. Records were based on the motif nucleotides and their reverse complementary sequence from the opposite strand. For example, the dinucleotide AG includes the motifs AG and GA, and the complements TC and CT; the trinucleotide GAA includes the motifs GAA, AAG and AGA and their complements CTT, TTC and TCT.

### Gene content and functional annotation

After masking for the occurrence of any kind of repetitive elements, sequences were compared to all records of the TIGR Plant Gene Indices [69] and NR database from NCBI, containing records of non-identical proteins from various databases, such as GenBank, RefSeq and UniProt. This was done using the BLASTX search tool [70] with a cutoff value of 1 × 10^−5^.

The functional annotation of the sequences was based on the BLAST2GO tools [71] for assigning ontological terms in accordance with BLASTX results, using 1×10^−6^ as the default annotation cutoff value. Gene Ontology (GO) is a controlled vocabulary that allows gene products from different species to be compared based on their annotations and the three independently organized gene ontologies, molecular function, biological process, and cellular component [72]. Vocabularies were reduced using the term plant, with the aid of a simplified version of the full ontologies (GOslim terms).

The count of GC bases and the estimate of GC content of the yellow passion fruit genome was performed by a PERL script (Practical Extraction and Report Language), developed in our laboratory. Percentage GC content was calculated according to the formula: .

### Microsynteny of genome regions

Areas of potential microsynteny were identified using the algorithm BWA-SW (Burrows-Wheeler Aligner’s Smith-Waterman Alignment) [73]. BWA is a software package for mapping low-divergent sequences against a large reference genome, and BWA-SW was designed for sequence reads ranging from 70 bp to 1,000 bp. Here, the BES sequences were compared to the complete genomic sequences of *Arabidopsis thaliana*, *Vitis vinifera* and *Populus trichocarpa*, downloaded from the Phytozome database [74]. The last two species are the closest *Passiflora edulis* relatives whose genomes have been annotated.

Each comparison, containing both the BAC-ends sequenced (forward and reverse sequences of the same BAC) was classified according to the position and orientation of the sequences. A particular region was considered to be potentially microsyntenic if it had been mapped to the same chromosome with a minimal distance between the paired matches of 15 kb and a maximum of 350 kb. Otherwise, the region was considered as rearranged. Manual evaluation of each alignment was performed using the IGV-Integrative Genomics Viewer v. 2.3 [75].

## Electronic supplementary material

Additional file 1: Table S1: Chromosome regions of three-reference genomes showing potential microsynteny with *Passiflora edulis* sequences. (DOC 58 KB)

## References

[1] Ulmer T, Macdougal J (2004). Passiflora: Passion Flowers of the World.

[2] Killip EP (1938). The American species of *Passifloraceae*. Publ Field Mus Nat Hist Bot.

[3] Feuillet C, Macdougal JM (2003). Checklist of recognized species names of passion flowers. Passiflora.

[4] Hansen AK, Gilbert LE, Simpson BB, Downie SR, Cervi AC, Jansen RK (2006). Phylogenetic relationships and chromosome number evolution in *Passiflora*. Syst Bot.

[5] Yotoko KSC, Dornelas MC, Togni PD, Fonseca TC, Salzano FM, Bonatto SL, Freitas LB (2011). Does variation in genome sizes reflect adaptive or neutral processes? New clues from *Passiflora*. PLoS One.

[6] Souza MM, Palomino G, Pereira MG, Viana AP (2004). Flow cytometric analysis of genome size variation in some *Passiflora* species. Hereditas.

[7] Oliveira EJ, Vieira MLC, Garcia AAF, Munhoz CF, Margarido GRA, Consoli L, Matta FP, Moraes MC (2008). An integrated molecular map of yellow passion fruit based on simultaneous maximum-likelihood estimation of linkage and linkage phases. J Am Soc Hortic Sci.

[8] Cerqueira-Silva CBM, Santos ESL, Vieira JGP, Mori GM, Jesus ON Corrêa R, Souza AP (2014). New microsatellite markers for wild and commercial species of *Passiflora* (*Passifloraceae*) and cross-amplification. Appl Plant Sci.

[9] Muschner V, Lorenz A, Cervi AC, Bonatto S, Souza-Chies T, Salzano F, Freitas L (2003). A first molecular analysis of *Passiflora* (*Passifloraceae*). Am J Bot.

[10] Yockteng RS, Nadot S (2004). Phylogenetic relationships among *Passiflora* species based on the Glutamine Synthase nuclear gene expressed in the chloroplast (*ncpGS*). Mol Phylogenet Evol.

[11] Cutri L, Dornelas MC (2012). PASSIOMA: Exploring expressed sequence tags during flower development in *Passiflora* spp. Comp Funct Genomics.

[12] Rudnicki M, Silveira MM, Pereira TV, Oliveira MR, Reginatto FH, Dal-Pizzol F, Moreira JCF (2007). Protective effects of *Passiflora alata* extract pretreatment on carbon tetrachloride induced oxidative damage in rats. Food Chem Toxicol.

[13] IBGE (2013). Produção agrícola municipal: culturas temporárias e permanentes 2011.

[14] Wing RA, Ammiraju JSS, Luo M, Kim H, Yu Y, Kudrna D, Goicoechea JL, Wang W, Nelson W, Rao K, Brar D, Mackill DJ, Han B, Soderlund C, Stein L, SanMiguel P, Jackson S (2005). The *Oryza* map alignment project: the golden path to unlocking the genetic potential of wild rice species. Plant Mol Biol.

[15] Bayou N, M’rad R, Belhaj A, Daoud H, Jemaa LB, Zemni R, Briault S, Helayem MB, Chaabouni H (2008). De novo balanced translocation t (7;16) (p22.1; p11.2) associated with autistic disorder. J Biomed Biotechnol.

[16] Wei F, Stein JC, Liang C, Zhang J, Fulton RS, Baucom RS, Paoli E, Zhou S, Yang L, Han Y, Pasternak S, Narechania A, Zhang L, Yeh C-T, Ying K, Nagel DH, Collura K, Kudrna D, Currie J, Lin J, Kim H, Angelova A, Scara G, Wissotski M, Golser W, Courtney L, Kruchowski S, Graves TA, Rock SM, Adams S (2009). Detailed analysis of a contiguous 22-Mb region of maize genome. PLoS Genet.

[17] Breen JM, Wicker T, Kong X, Zhang J, Ma W, Paux E, Feuillet C, Appels R, Bellgard M (2010). A highly conserved gene island of three genes on chromosome 3B of hexaploid wheat: diverse gene function and genomic structure maintained in a tightly linked block. BMC Plant Biol.

[18] Paiva JAP, Prat S, Vautrin S, Santos MD, San-Clemente H, Brommonschenkel S, Fonseca PGS, Grattapaglia D, Song X, Ammiraju JSS, Kudrna D, Wing RA, Freitas AT, Bergès H, Grima-Pettenati J (2011). Advancing *Eucalyptus* genomics: identification and sequencing of lignin biosynthesis genes from deep-coverage BAC libraries. BMC Genomics.

[19] Budiman MA, Mao L, Wood TC, Wing RA (2000). A deep-coverage tomato BAC library and prospects toward development of an STC framework for genome sequencing. Genome Res.

[20] Wu C, Sun S, Nimmakayala P, Santos FA, Meksem K, Springman R, Ding K, Lightfoot DA, Zhang HB (2004). A BAC and BIBAC-based physical map of the soybean genome. Genome Res.

[21] Ammiraju JS, Luo M, Goicoechea JL, Wang W, Kudrna D, Mueller C, Talag J, Kim H, Sisneros NB, Blackmon B, Fang E, Tomkins JB, Brar D, MacKill D, McCouch S, Kurata N, Lambert G, Galbraith DW, Arumuganathan K, Rao K, Walling JG, Gill N, Yu Y, SanMiguel P, Soderlund C, Jackson S, Wing RA (2006). The *Oryza* bacterial artificial chromosome library resource: Construction and analysis of 12 deep-coverage large-insert BAC libraries that represent the 10 genome types of the genus *Oryza*. Genome Res.

[22] Hu Y, Lu Y, Ma D, Guo W, Zhang T (2011). Construction and characterization of a bacterial artificial chromosome library for the A-genome of cotton (*G. arboreum* L.). J Biomed Biotechnol.

[23] Figueira TRSF, Okura V, Silva FR, Silva MJ, Kudma D, Ammiraju JSS, Taag J, Wing RA, Arruda P (2012). A BAC library of the SP80-3280 sugarcane variety (*Saccharum* sp.) and its inferred microsynteny with the sorghum genome. BMC Res Notes.

[24] Shultz JL, Kazi S, Bashir R, Afzal JA, Lightfoot DA (2007). The development of BAC-end sequence-based microsatellite markers and placement in the physical and genetic maps of soybean. Theor Appl Genet.

[25] Han Y, Korban SS (2008). An overview of the apple genome through BAC-end sequence analysis. Plant Mol Biol.

[26] Cavagnaro PF, Chung SM, Szklarczyk M, Grzebelus D, Senalik D, Atkins AE, Simon PW (2009). Characterization of a deep-coverage carrot (*Daucus carota* L.). BAC library and initial analysis of BAC-end sequences. Mol Genet Genomics.

[27] Rampant PF, Lesur I, Boussardon C, Bitton F, Martin-Magniette M, Bodenes C, Le Provost G, Berges H, Fluch S, Kremer A, Plomion C (2011). Analysis of BAC end sequences in oak, a keystone forest tree species, providing insight into the composition of its genome. BMC Genomics.

[28] Lin J, Davekudrna I, Wing RA (2011). Construction, characterization, and preliminary BAC-end sequence analysis of a bacterial artificial chromosome library of the tea plant (*Camellia sinensis*). J Biomed Biotechnol.

[29] Magbanua ZV, Ozkan S, Bartlett BD, Chouvarine P, Saski CA, Liston A, Cronn RC, Nelson CD, Peterson DG (2011). Adventures in the enormous: a 1.8 million clone BAC library for the 21.7 Gb genome of loblolly pine. PLoS One.

[30] Cuco SM, Vieira MLC, Mondin M, Aguiar-Perecin MLR (2005). Comparative karyotype analysis of three Passiflora L. species and cytogenetic characterization of somatic hybrids. Caryologia.

[31] Praça MM, Carvalho RC, Marcelino FC, Mendonça MAC (2008). Morphological aspects of Passiflora edulis f. flavicarpa chromosomes using acridine orange banding and rDNA-FISH tools. Caryologia.

[32] Penha HP (2012). Construção de uma biblioteca genômica de Passiflora edulis f. flavicarpa inserida em BACs (Bacterial Artificial Chromosome) e mapeamento cromossômico usando hibridação in situ fluorescente.

[33] Jaillon O, Aury JM, Noel B, Policriti A, Clepet C, Casagrande A, Choisne N, Aubourg S, Vitulo N, Jubin C, Vezzi A, Legeai F, Hugueney P, Dasilva C, Horner D, Mica E, Jublot D, Poulain J, Bruyère C, Billault A, Segurens B, Gouyvenoux M, Ugarte E, Cattonaro F, Anthouard V, Vico V, Fabbro CD, Alaux M, Gaspero GD, Dumas V (2007). The grapevine genome sequence suggests ancestral hexaploidization in major angiosperm phyla. Nature.

[34] Tuskan GA, Difazio S, Jansson S, Bohlmann J, Grigoriev I, Hellsten U, Putnam N, Ralph S, Rombauts S, Salamov A, Schein J, Sterck L, Aerts A, Bhalerao RR, Bhalerao RP, Blaudez D, Boerjan W, Brun A, Brunner A, Busov V, Campbell M, Carlson J, Chalot M, Chapman J, Chen G-L, Cooper D, Coutinho PM, Couturier J, Covert S, Cronk Q (2006). The genome of black cottonwood, *Populus trichocarpa* (Torr. & Gray). Science.

[35] Chan AP, Crabtree J, Zhao Q, Lorenzi H, Orvis J, Puiu D, Melake-Berhan A, Jones KM, Redman J, Chen G, Cahoon EB, Gedil M, Stanke M, Haas BJ, Wortman JR, Fraser-Liggett CM, Ravel J, Rabinowicz PD (2010). Draft genome sequence of the oilseed species *Ricinus communis*. Nat Biotechnol.

[36] Kuhl JC, Cheung F, Yuan Q, Martin W, Zewdie Y, McCallum J, Catanach A, Rutherford P, Sink KC, Jenderek M, Prince JP, Town CD, Havey MJ (2004). A unique set of 11,008 onion expressed sequence tags reveals expressed sequence and genomic differences between the monocot orders Asparagales and Poales. Plant Cell.

[37] Munhoz CF (2012). Identificação de genes de maracujá azedo diferencialmente expressos durante a interação com Xanthomonas axonopodis.

[38] Lai CW, Yu Q, Hou S, Skelton RL, Jones MR, Lewis KL, Murray J, Eustice M, Guan P, Agbayani R, Moore PH, Ming R, Presting GG (2006). Analysis of papaya BAC end sequences reveals first insights into the organization of a fruit tree genome. Mol Genet Genomics.

[39] The Arabidopsis Initiative (2000). Analysis of the genome sequence of the flowering plant *Arabidopsis thaliana*. Nature.

[40] González VM, Rodríguez-Moreno L, Centeno E, Benjak A, Garcia-Mas J, Puigdomènech P, Aranda MA (2010). Genome-wide BAC-end sequencing of *Cucumis melo* using two BAC libraries. BMC Genomics.

[41] Wu J, Gu YQ, Hu Y, You FM, Dandekar AM, Leslie CA, Aradhya M, Dvorak J, Luo MC (2012). Characterizing the walnut genome through analyses of BAC end sequences. Plant Mol Biol.

[42] Terol J, Naranjo MA, Ollitrault P, Talon M (2008). Development of genomic resources for *Citrus clementina*: characterization of three deep-coverage BAC libraries and analysis of 46,000 BAC end sequences. BMC Genomics.

[43] Hsu CC, Chung YL, Chen TC, Lee YL, Kuo YT, Tsai WC, Hsiao YY, Chen YW, Wu WL, Chen HH (2011). An overview of the *Phalaenopsis* orchid genome through BAC end sequence analysis. BMC Plant Biol.

[44] Cheung F, Town CD (2007). A BAC end view of the *Musa acuminata* genome. BMC Plant Biol.

[45] Velasco R, Zharkikh A, Affourtit J, Dhingra A, Cestaro A, Kalyanaraman A, Fontana P, Bhatnagar SK, Troggio M, Pruss D, Salvi S, Pindo M, Baldi P, Castelletti S, Cavaiuolo M, Coppola G, Costa F, Cova V, Dal Ri A, Goremykin V, Komjanc M, Longhi S, Magnago P, Malacarne G, Malnoy M, Micheletti D, Moretto M, Perazzolli M, Si-Ammour A, Vezzulli S (2010). The genome of the domesticated apple (*Malus* × *domestica* Borkh.). Nat Genet.

[46] Ming R, Yu Q, Moore PH, Paull RE, Chen NJ, Wang M-L, Zhu YJ, Schuler MA, Jiang J, Paterson AH (2012). Genome of papaya, a fast growing tropical fruit tree. Tree Genet Genomics.

[47] Vicient CM, Jääskeläinen M, Kalendar R, Schulman AH (2001). Active retrotransposons are a common feature of grass genomes. Plant Physiol.

[48] McCarthy EM, Liu J, Lizhi G, McDonald JF (2002). Long terminal repeat retrotransposons of *Oryza sativa*. Genome Biol.

[49] Zhao M, Ma J (2013). Co-evolution of plant LTR retrotransposons and their host genomes. Protein Cell.

[50] Kim C, Lee TH, Compton RO, Robertson JS, Pierce GJ, Paterson AH (2013). A genome-wide BAC end-sequence survey of sugarcane elucidates genome composition, and identifies BACs covering much of the euchromatin. Plant Mol Biol.

[51] International Rice Genome Sequencing Project (2005). The map based sequence of the rice genome. Nature.

[52] Ingvarsson PK (2005). Nucleotide polymorphism and linkage disequilibrium within and among natural populations of European aspen (*Populus tremula* L., Salicaceae). Genetics.

[53] Pereira GS, Nunes ES, Laperuta LC, Braga MF, Penha HA, Diniz AL, Munhoz CF, GAZAFFI R, Garcia AAF, Vieira MLC (2013). Molecular polymorphism and linkage analysis in sweet passion fruit, an outcrossing species. Ann Appl Biol.

[54] Hong CP, Plaha P, Koo D, Yang T, Choi SR, Lee YK, Uhm T, Bang J, Edwards D, Bancroft I, Park B, Lee J, Lim YP (2006). A survey of the *Brassica rapa* genome by BAC-end sequence analysis and comparison with *Arabidopsis thaliana*. Mol Cells.

[55] Datema E, Mueller LA, Buels R, Giovannoni JJ, Visser RGF, Stiekema WJ, van Ham RCGJ (2008). Comparative BAC end sequence analysis of tomato and potato reveals overrepresentation of specific gene families in potato. BMC Plant Biol.

[56] Anwar T, Khan AU (2005). Mapping and analysis of simple sequence repeats in the *Arabidopsis thaliana* genome. Bioinformation.

[57] CNRGV: *The French Plant Genomic Resource Center*. [http://cnrgv.toulouse.inra.fr/]

[58] Peterson DG, Tomkins JP, Frisch DA, Wing RA, Paterson AH (2000). Construction of plant bacterial artificial chromosome (BAC) libraries: An illustrated guide. J Agric Genomics.

[59] Gonthier L, Bellec A, Blassiau C, Prat E, Helmstetter N, Rambaud C, Huss B, Hendriks T, Bergès H, Quillet M-C (2010). Construction and characterization of two BAC libraries representing a deep-coverage of the genome of chicory (*Cichorium intybus* L., *Asteraceae*). BMC Res Notes.

[60] Cabral JS, Felix LP, Guerra M (2006). Heterochromatin diversity and its co-localization with 5S and 45S rDNA sites in chromosomes of four *Maxillaria* species (Orchidaceae). Genet Mol Biol.

[61] Barros Silva AE, Marques A, Santos KGB, Guerra M (2010). The evolution of CMA bands in *Citrus* and related genera. Chromosome Res.

[62] Pedrosa A, Sandal N, Stougaard J, Schweizer D, Bachmair A (2002). Chromosomal map of the model legume *Lotus japonicus*. Genetics.

[63] Zwick MS, Hanson RE, McKnight TD, Islam-Faridi MN, Stelly DM, Wing RA, Price HJ (1997). A rapid procedure for isolation of C_o_*t*-1 DNA from plants. Genome.

[64] Heslop-Harrison JS, Harrison GE, Leitch IJ (1992). Reprobing of DNA: DNA *in situ* hybridization preparations. Trends Genet.

[65] Ewing B, Hillier L, Wendl MC, Green P (1998). Base-calling of automated sequencer traces using PHRED. I. Accuracy assessment. Genome Res.

[66] Durham AM, Kashiwabara AY, Matsunaga FT, Ahagon PH, Rainone F, Varuzza LE, Gruber A (2005). EGene: a configurable pipeline generation system for automated sequence analysis. Bioinformatics.

[67] Ouyang S, Buell CR (2004). The TIGR Plant Repeat Databases: a collective resource for the identification of repetitive sequences in plants. Nucleic Acids Res.

[68] Kofler R, Shlötterer C, Lelley T (2007). SciRoKo: A new tool for whole genome microsatellite search and investigation. Bioinformatics.

[69] Quackenbush J, Liang F, Holt I, Pertea G, Upton J (2000). The TIGR gene indices: reconstruction and representation of expressed gene sequences. Nucleic Acids Res.

[70] Altschul SF, Madden TL, Schäffer AA, Zhang J, Zhang Z, Miller W, Lipman DJ (1997). Gapped BLAST and PSI-BLAST: a new generation of protein database search programs. Nucleic Acids Res.

[71] Conesa A, Götz S, Garcia-Gomez JM, Terol J, Talon M, Robles M (2005). Blast2GO: a universal tool for annotation, visualization and analysis in functional genomics research. Bioinformatics.

[72] Ashburner M, Ball CA, Blake JA, Botstein D, Butler H, Cherry JM, Davis AP, Dolinski K, Dwight SS, Eppig JT, Harris MA, Hill DP, Issel-Tarver L, Kasarskis A, Lewis S, Matese JC, Richardson JE, Ringwald M, Rubin GM, Sherlock G (2000). Gene ontology: tool for the unification of biology. The Gene Ontology Consortium. Nat Genet.

[73] Durbin R, Li H (2010). Fast and accurate long-read alignment with Burrows-Wheeler transform. Bioinformatics.

[74] Goodstein DM, Shu S, Howson R, Neupane R, Hayes RD, Fazo J, Mitros T, Dirks W, Hellsten U, Putnam N, Rokhsar DS (2012). Phytozome: a comparative platform for green plant genomics. Nucleic Acids Res.

[75] Thorvaldsdóttir H, Robinson JT, Mesirov JP (2013). Integrative Genomics Viewer (IGV): high-performance genomics data visualization and exploration. Brief Bioinform.

