# Supplementary material for: Begin at the beginning: A BAC-end view of the passion fruit (Passiflora) genome
Source: BMC Genomics. 2014 Sep 26;15(1):816. doi: 10.1186/1471-2164-15-816 (PMC4189760; doi:10.1186/1471-2164-15-816)
Supplement: Supplementary file 1 — Additional file 1: Table S1: Chromosome regions of three-reference genomes showing potential microsynteny with Passiflora edulis sequences. (DOC 58 KB) [file 12864_2014_6494_MOESM1_ESM.doc]

**Additional file 1**: **Table S1:** Chromosome regions of three-reference genomes showing potential microsynteny with *Passiflora edulis* sequences

| **Paired end sequences (F/R)** | **Genome of reference** | **Coordinates (F/R)** | **Span (in kb)** | **Category** | **Gene content** |
| --- | --- | --- | --- | --- | --- |
| Pe85Q4F4 | *Arabidopsis thaliana* chromosome C | 15,558-107,835 | 92.28 | Collinear | **58** |
| Pe69Q4G9 |  | 50,130-130,159 | 80.03 | Collinear | **55** |
| Pe164Q1G7 |  | 72,358-106,018 | 33.66 | Rearranged |  |
| Pe164Q2A6 | *Vitis vinifera* chromosome 11 | 2,324,226-2,502,417 | 178.19 | Collinear | **26** |
| Pe164Q1G7 |  | 10,252,239-10,261,324 | 9.08 | Rearranged |  |
| Pe69Q4G9 | *V. vinifera* chromosome 15 | 5,395,003-9,753,677 | 4,358.67 | Gapped |  |
| Pe85Q4F4 | *V. vinifera* chromosome 10/5 |  |  | Non co-localized |  |
| Pe214Q2A9 | *V. vinifera* chromosome 16/1 |  |  | Non co-localized |  |
| Pe216Q4A11 | *Populus trichocarpa* chromosome 12 | 9,673,965-9,890,993 | 217.03 | Collinear | **21** |
| Pe75Q4B6 | *P. trichocarpa* chromosome 14 | 9,166,211-9,343,042 | 176.83 | Collinear | **29** |
| Pe164Q1F9 |  | 5,539,303-5,845,525 | 306.22 | Collinear | **45** |
| Pe173Q4A8 | *P. trichocarpa* chromosome 9 | 10,462,257-10,657,680 | 195.42 | Rearranged |  |
| Pe164Q1G7 | *P. trichocarpa* chromosome 11 | 7,197,221-13,862,010 | 6,664.79 | Gapped |  |
| Pe164Q2A6 | *P. trichocarpa* chromosome 18/6 |  |  | Non co-localized |  |
| Pe1Q1G9 | *P. trichocarpa* chromosome 5/2 |  |  | Non co-localized |  |
| Pe214Q2A9 | *P. trichocarpa* chromosome 12/15 |  |  | Non co-localized |  |
| Pe93Q2D2 | *P. trichocarpa* chromosome 8/10 |  |  | Non co-localized |  |
| Pe93Q4D8 | *P. trichocarpa* chromosome 4/ scaffold 21 |  |  | Non co-localized |  |
| Pe216Q1E3 | *P. trichocarpa* chromosome 6/18 |  |  | Non co-localized |  |
| Pe141Q2F4 | *P. trichocarpa* chromosome 3/1 |  |  | Non co-localized |  |
| Pe69Q1B4 | *P. trichocarpa* chromosome 9/16 |  |  | Non co-localized |  |
| Pe69Q1C6 | *P. trichocarpa* scaffold 104/ chromosome 10 |  |  | Non co-localized |  |
| Pe69Q4G9 | *P. trichocarpa* chromosome 11/13 |  |  | Non co-localized |  |
| Pe75Q1F8 | *P. trichocarpa* chromosome 4/17 |  |  | Non co-localized |  |
| Pe214Q3D6 | *P. trichocarpa* chromosome 9/4 |  |  | Non co-localized |  |
